# Identifying Opportunities for Prevention of Adverse Outcomes Following Female Genital Fistula Repair: Protocol for a Mixed-Methods Study in Uganda

**DOI:** 10.21203/rs.3.rs-2879899/v1

**Published:** 2023-05-05

**Authors:** Alison M El Ayadi, Susan Obore, Fred Kirya, Suellen Miller, Abner Korn, Hadija Nalubwama, John Neuhaus, Monica Getahun, Patrick Eyul, Robert Twine, Erin V. W. Andrew, Justus K. Barageine

**Affiliations:** University of California, San Francisco; Mulago Specialized Women and Neonatal Hospital; Soroti University; University of California, San Francisco; University of California, San Francisco; Makerere University; University of California, San Francisco; University of California, San Francisco; Infectious Disease Research Collaboration; Infectious Disease Research Collaboration; Thomas Jefferson University; Makerere University

**Keywords:** Female genital fistula, vesicovaginal fistula, obstructed labor, stillbirth, fistula repair, reconstructive surgery, recurrence, reintegration, post-repair incontinence, mixed-methods

## Abstract

**Background.:**

Female genital fistula is a traumatic debilitating injury, frequently caused by prolonged obstructed labor, affecting between 500,000–2 million women in lower-resource settings. Vesicovaginal fistula causes urinary incontinence. Other gynecologic, neurologic and orthopedic morbidity may occur during fistula development. Women with fistula are stigmatized; limit engagement in social, economic, or religious activities; and report high psychiatric morbidity. Improved global surgical access has reduced fistula consequences yet post-repair risks impacting quality of life and well-being include fistula repair breakdown or recurrence and ongoing or changing urine leakage or incontinence. Limited evidence on risk factors contributing to adverse outcomes hinders interventions to mitigate adverse events, protecting health and quality of life after surgery. This study seeks to identify predictors and characteristics of post-repair fistula breakdown and recurrence (Aim 1) and post-repair incontinence (Aim 2), and to identify feasible and acceptable intervention strategies (Aim 3).

**Methods.:**

This mixed-methods study incorporates a prospective cohort study of women with successful vesicovaginal fistula repair at approximately 12 fistula repair centers and affiliated care sites in Uganda (Aims 1–2) followed by qualitative inquiry among key stakeholders (Aim 3). Cohort participants will have a baseline visit at the time of surgery followed by data collection at 2 weeks, 6 weeks, 3 months and quarterly thereafter for 3 years. Primary predictors to be evaluated include patient-related factors, fistula-related factors, fistula repair-related factors, and post-repair behaviors and exposures, collected via structured questionnaire at all data collection points. Clinical exams will be conducted at baseline, 2 weeks post-surgery, and for outcome confirmation at symptom development. Primary outcomes are fistula repair breakdown or fistula recurrence and post-repair incontinence. In-depth interviews will be conducted with cohort participants (n ~ 40) and other key stakeholders (~ 40 including family, peers, community members and clinical/social service providers) to develop feasible and acceptable intervention concepts for adjustment of identified risk factors.

**Discussion.:**

Participant recruitment is underway. This study is expected to identify key predictors that can directly improve fistula repair and post-repair programs and women’s outcomes, optimizing health and quality of life. Furthermore, our study will create a comprehensive longitudinal dataset capable of supporting broad inquiry into post-fistula repair health.

**Trial Registration.:**

ClinicalTrials.gov Identifier: NCT05437939

## Plain English Summary

Female genital fistula is a traumatic birth injury which occurs where access to emergency childbirth care is poor. It causes uncontrollable urine leakage and is associated with other physical and psychological symptoms. Due to the urine leakage and its odor, women with fistula are stigmatized which has mental health and economic consequences. Ensuring women’s access to fistula surgery and ongoing wellbeing is important for limiting the impact of fistula. After fistula surgery, health risks such as fistula repair breakdown or recurrence or changes to urine leakage can happen, but studies during this time are limited. Our study seeks to measure these health risks and factors influencing these risks quantitatively, and work with patients, community members, and fistula care providers to come up with solutions. We will recruit up to 1000 participants into our study at the time of fistula surgery and follow them for three years. We will collect patient and clinical data through patient survey and medical record review. If participants have changes in urine leakage, they will be asked to return to the fistula repair hospital for exam. We will interview about 80 individuals to obtain their ideas for feasible and acceptable intervention options. We expect that this study will help to understand risk factors for poor health following fistula repair and, eventually, improve women’s health and quality of life after fistula.

## Background

Female genital fistula is a traumatic debilitating injury affecting somewhere between 500,000 to 2 million women, mostly in sub-Saharan Africa, with up to 100,000 annual incident cases.^1,2^ Most often caused by prolonged obstructed labor, other etiologies are iatrogenic or traumatic. Many births resulting in fistula end in stillbirth.^3^ Women with fistula experience uncontrollable urinary and/or fecal leakage through the vagina.^4,5^ Other consequences of obstructed labor injury complex include neurologic injury, gynecologic morbidity, and orthopedic trauma, resulting in pain, weakness, difficulty walking and secondary infertility.^6,7^ Women with fistula are stigmatized, which restricts their participation in social and economic activities,^3,6^ and report substantial psychiatric morbidity.^8–10^

Although improved global surgical access has begun to reduce the significant physical, psychosocial, and economic consequences of fistula, women with successful surgery may continue to face adverse experiences or events following surgery. Research estimates the incidence of surgical repair breakdown or post-repair fistula recurrence in 2.1%–18.4% of women following surgery, largely within the first 12 months.^11–15^ Post-repair incontinence continues among about one-third of women despite closed fistula,^16–18^ with some individuals developing incident incontinence despite initial post-surgical resolution.^14,15^ Fistula recurrence, urinary and fecal incontinence are associated with significant ongoing physical and psychological disability. From the patient perspective, fistula closure and persistent incontinence are both associated with ongoing urinary leakage and can have similar repercussions including ongoing psychiatric morbidity and physical discomfort,^6,8–10,19−22^ enacted and anticipated stigma causing social isolation, depressive symptoms and continued social and economic consequences.^23–27^

The evidence base regarding persistent post-repair risks and consequences is underdeveloped, which limits clinical and counseling interventions capable of mitigating harmful processes. Most evaluation has been retrospective. Most previous prospective research on repair failure is limited to the fistula repair hospitalization or early clinical-follow up, with sparse data on predictors of later fistula repair breakdown and recurrence. A study in Guinea reported that women not sexually active at follow-up and those with urethral involvement, damaged bladder neck, and presence of vaginal scarring had significantly higher incidence of fistula recurrence.^14^ Self-reported precursors to fistula recurrence in this study included farm work (19%), walking (12%), sexual intercourse (10%), and pregnancy or childbirth (10%).^14^ In Ethiopia, recurrence was linked to physical strain, sexual intercourse, heavy work, or jostling during transit.^15^ Three smaller studies in Ethiopia, Malawi, and Sudan identified fistula recurrences occurring during a subsequent childbirth.^12,28,29^

Fistula recurrence can be mechanistically characterized as surgical breakdown or re-injury, thus capture of the biological and social factors important to each potential pathway is key. The literature on enhanced recovery after surgery has identified patient counseling, physical conditioning, avoidance of alcohol and smoking, and good nutrition as protective.^30^ Surgical site infection, an intermediate factor associated with late breakdown, is more common among patients with co-morbidities (particularly diabetes), advanced age, risk indices, and lengthier surgery.^31^ Factors responsible for re-injury may include the biological and social structures that increased women’s risk of developing a first fistula, including limited access to emergency obstetric care, and it is possible that a woman’s risk of fistula recurrence following surgery will be different due to the biological alterations occurring within the surgery itself, largely impacted by fistula characteristics which are likely to be important predictors of recurrence outcomes. A small Ethiopian study comparing women with and without persistent post-repair urinary incontinence found that leakage was significantly more likely among younger women at first vaginal birth.^22^

Urinary incontinence following fistula repair is complex due to a variety of anatomic and functional factors at play which often remain uncharacterized, particularly over time.^32–34^ Research on potential intervention points for reducing persistent and incident post-repair incontinence is limited by the breadth of factors assessed, lack of differentiation between incontinence types, and lack of longitudinal follow-up. Predictors of immediate post-repair incontinence include fistula severity, including size and location, presence of vaginal scarring, and shorter urethral length.^11,16,35^ Less is known about incident incontinence following successful surgical resolution.^14,17^

To inform clinical and counseling interventions to optimize women’s health and quality of life following fistula repair, we have developed a mixed-methods research study incorporating a longitudinal cohort to robustly identify predictors of fistula repair breakdown and recurrence (Aim 1), identify predictors and characteristics of post-repair incontinence (Aim 2), supplemented by qualitative work among key stakeholders to identify feasible and acceptable strategies for modifying key risk factors of adverse outcomes (Aim 3). The three proposed specific aims address critical research gaps in the fistula literature and are expected to tangibly inform the development of clinical and counseling interventions to mitigate complications, improve post-surgical outcomes and quality of life. Our investigative approach seeks to elucidate which factors (e.g., patient and fistula characteristics, surgical factors, and post-repair behaviors and exposures) are most important in determining risk of post-surgical adverse outcome, thereby leading to appropriately targeted and contextually-adapted interventions, and will identify priority research areas needed for women with continued poor outcomes.

## Methods

**Figure 1:**
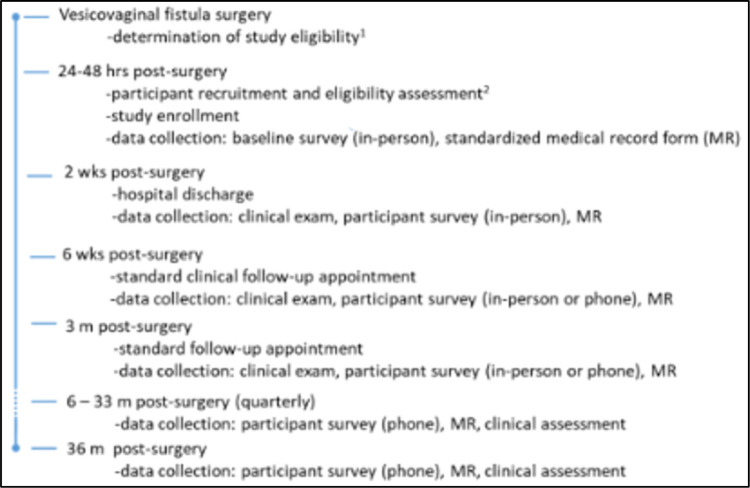
Overview of study data collection and timeline

**Figure 2:**
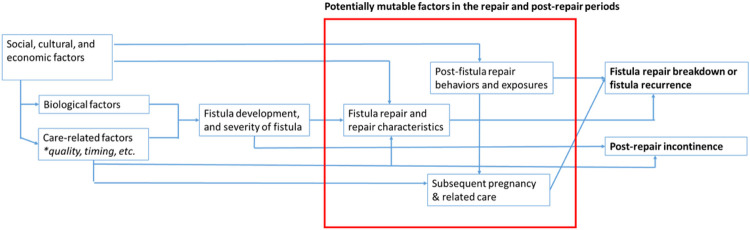
Summarized conceptual framework between predictors and adverse fistula outcomes

This sequential explanatory mixed-methods study incorporates a longitudinal cohort which will enroll and follow up to 1000 Ugandan women with successfully closed genital fistula from the time of fistula surgery through 36-months post-surgery (quantitative) and in-depth interviews which will be conducted with ~ 80 purposively selected key stakeholders for translation of quantitative findings into feasible and acceptable interventions (Fig. 1). The summarized conceptual framework guiding our study (Fig. 2) highlights factors to be explored including both invariable and potentially mutable factors to develop the evidence base that will allow them to maintain their reproductive and overall health over time.

### Study setting and sites

Our research is situated in Uganda which reports a lifetime fistula symptom prevalence of 19.2 per 1,000 reproductive aged women and ~ 5,000 new cases annually.^36–38^ Uganda’s National Fistula Technical Working Group (est. 2002) with representatives from the Ministry of Health, international and national non-governmental organizations (NGOs), medical professionals, and media has focused on increasing fistula surgery availability; from 2010 to 2015 the annual number of fistula surgeries in Uganda increased from 1377 to 2065.^39^ Fistula surgery is available at 20 centers of excellence in Uganda, with 25 trained surgeons with various levels of experience employed by national and regional referral hospitals. Regional literature suggests that genital fistula repair is successful among ~ 80% of affected women.^18,40^

**Figure 3:**
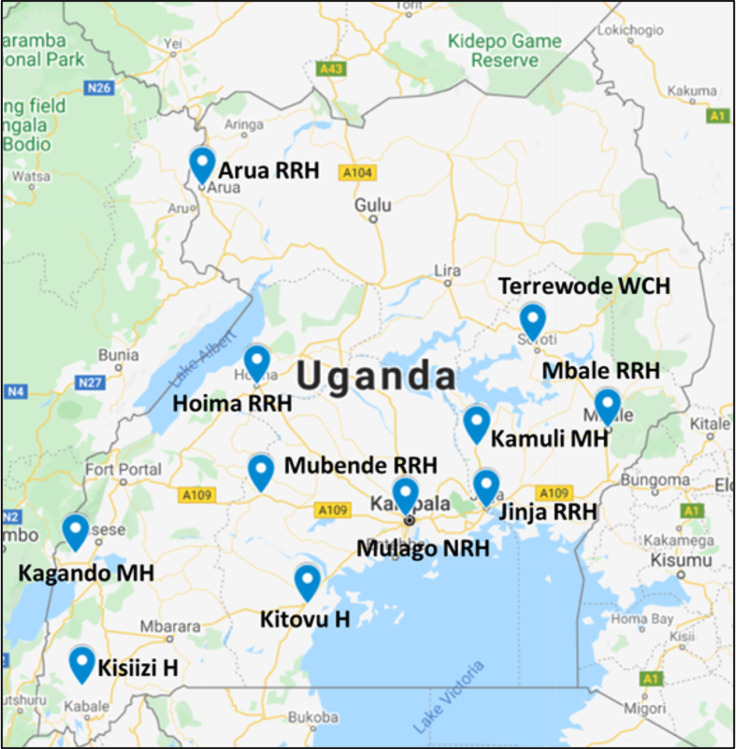
Partnering fistula repair study sites across Uganda

The research team is partnering with approximately 12 facilities across Uganda providing fistula repair, selected for fistula repair volume, geographic proximity, prior collaboration and research capability (Fig. 3). Various fistula repair models are used across these sites; some sites conduct routine surgeries within ongoing urogynecological services only, others conduct fistula repair camps only, and others combine both routine care and camp models. Patients who are under the care of our study providers and research assistants at alternative inconsistent locations will be considered eligible for study participation if they meet study eligibility criteria.

### Longitudinal cohort study

Our study will recruit a longitudinal cohort of 1,000 women with successful (closed) vesicovaginal fistula repair just after surgery (~ 48 hours later). Participants will be followed for 3 years in total, with data collected via questionnaire at baseline (surgery), 2 weeks (hospital discharge), 6 weeks, 3 months and quarterly thereafter.

#### Study participants.

Inclusion criteria are vesicovaginal fistula, completed fistula surgery with confirmed closure, age 15 or above (where individuals 15–17 meet Ugandan legal criteria for emancipation), and capable and willing to provide informed consent. Exclusion criteria are do not live within a feasible location for follow-up, operationalized by return travel back to fistula repair facility and cellular telephone network.

In the case of fresh fistula, we allow for a special circumstance for study participant inclusion criteria: If limited in size (< 2cm) and time since occurrence (< 3 months), catheterization alone may successfully heal ~ 10% of fistula.^41^ Where a potential participant’s fistula is considered eligible for treatment via catheterization instead of surgery, and the participant undergoes catheterization and is cured (defined as fistula closure, confirmed by methylene blue test), this woman will be considered eligible for study enrollment if she meets all other study eligibility criteria except having undergone surgery.

#### Study procedures.

Local study researchers will recruit participants into the cohort following confirmation of successful fistula repair. Potentially eligible women will be identified through review of urogynecology department surgical logbooks, patient medical records, and via direct communication with fistula surgeons and other providers. After fistula repair, women stay at the repair facility for 14 days at a minimum for post-repair catheterization. The local study researcher will approach women at the facility who meet the eligibility criteria in-person 24–48 hrs after surgery to explain the study to them, assess whether they are eligible and, if so, invite them to participate. The study researcher will complete the full informed consent procedure for those women who indicate that they would like to participate, at a convenient time, with the use of a decision tool developed by the University of California San Francisco Human Subjects Research Ethics Board. The study researcher will then collect participant contact information and administer the baseline questionnaire. Participants will be followed through 36 months post-repair, regardless of outcome.

Two-week data collection will occur prior to hospital discharge. Other planned follow-up data collection (6 weeks and quarterly, starting at 3 months) will occur over mobile phone, given high mobile phone penetration across Uganda.^42,43^ Where women do not have their own phone, the study will provide them with a study phone and phone number. Airtime will be provided throughout the study to ensure that study participants have call time (minutes) available and could pay to charge their phone battery as needed.

Clinical exams will be conducted at baseline and 2 weeks post-surgery for fistula and repair characterization, and as necessary based on women’s self-report of symptom development across the study follow-up to validate outcomes. Participant questionnaires will include a series of signs and symptoms which, if reported, will trigger clinical evaluation at the repair facility for determination of study outcomes: *de novo* urinary or fecal incontinence signifying fistula repair breakdown and recurrence, or any change in urinary incontinence. Women reporting such symptoms on interviewer-administered questionnaire or through other study or clinical communication will return to the fistula repair facility for outcome assessment and clinical care following standard clinical procedures. Clinical assessment is routinely conducted at the in-person follow-up appointments at 6 weeks and 3 months post-repair. Transportation costs will be reimbursed for all follow-up data collection required.

It is possible that some participants may require multiple fistula surgeries during the course of their study participation. If this occurs, participant follow-up will be adjusted to incorporate data collection at 6 weeks and 3 months following the subsequent surgery, after which the regular quarterly data collection will continue through the originally targeted 36 months after the participant’s initial enrollment.

#### Measures.

Primary predictors to be investigated include patient-related factors, fistula-related factors, fistula repair-related factors, and post-repair behaviors and exposures (Table 1), collected via structured questionnaire at all follow-up data collection points and from medical records and clinical forms when examinations are performed. Participant sociodemographic, fistula and fistula-repair characteristics will be measured at baseline, with questions on post-repair behaviors and exposures at each follow-up. Outcomes will be screened for at each data collection point; study interviewers will ask a series of questions on signs and symptoms to identify outcomes of interest, women who report new or changed urinary incontinence will be asked to return to the fistula repair facility for clinical exam to confirm fistula breakdown, recurrence, or incontinence. Women with persistent incontinence without fistula breakdown/recurrence will be asked to respond to a short supplemental questionnaire to characterize this incontinence. Women identified as pregnant will be asked to respond to a short supplemental questionnaire on pregnancy-related exposures and outcomes. We seek to develop a comprehensive longitudinal dataset capable of supporting broad inquiry into health and wellbeing following fistula repair.

#### Data analysis.

To identify predictors of post-repair fistula breakdown and recurrence (Aim 1), we will first calculate the incidence of post-repair fistula breakdown and recurrence and its 95% confidence interval (CIs) overall by dividing the number of events identified by the total person-time observed. The probability of event-free survival at defined time points will be calculated using the Kaplan-Meier estimate. We will then estimate the individual and joint-effects of the patient, fistula, fistula repair, and post-repair characteristics on time to post-repair fistula breakdown and recurrence in order to identify significant factors in time to post-repair fistula breakdown and recurrence. We will fit proportional hazards frailty survival models to jointly analyze times to post-repair fistula breakdown and recurrence.^44^ These models will include a shared frailty at the subject level to accommodate within-subject correlation of times to breakdown and recurrence events and interactions of predictors with event type to accommodate potential differences in the association of predictors with times to the events. These models will also include a shared frailty at the provider level since patients will be clustered within providers within facilities. We will fit the frailty survival models using routines in Stata statistical analysis software.^45^

Prior to fitting multivariable models, we will calculate the estimated correlation of all potential predictors to identify any highly correlated groups of predictors. We will not include such groups of predictors in any multivariable models. We will assess the adequacy of the proportional hazards assumption through inspection of Schoenfield residuals as a function of time. In the event our data violate the proportional hazards assumption, we will modify our modelling approach to accommodate interactions or stratification, as is most appropriate for the data. We will subsequently fit one multivariable proportional hazards regression model to document the comparative relationship between patient, fistula, fistula repair, and post-repair characteristics and the hazard rate of post-repair fistula breakdown and recurrence integrating all independent variables that were associated with the outcome in bivariable analyses at a conservative p threshold of p < 0.1. Final model selection will be determined via Akaike’s Information Criteria.^52^ Secondary analyses will assess time to post-repair fistula breakdown (< 3 months post-repair) and time to fistula recurrence (≥ 3 months post-repair) separately, and by fistula etiology (obstetric versus iatrogenic), although our study is not powered for secondary outcomes.

Other methods will also be used to better understand the contribution of risk factors of fistula repair breakdown and recurrence. To overcome the biases inherent to observational research in understanding causal effects,^46^ we propose conducting a series of secondary analyses employing propensity score methods.^47^ Propensity score methods account for systematic differences in baseline characteristics between exposed and unexposed participants, allowing for effects to be interpreted as causal, similar to a randomized experiment.^48,49^ Indeed, propensity score methods may reduce systematic differences between treatment groups compared to covariate adjustment methods.^47^ For these analyses, we will estimate a series of models for each key modifiable factor to be assessed, first developing models predicting the probability of the particular exposure using key baseline and other measures deemed to be relevant for developing the treatment weight, followed by analyses of the exposure and outcome incorporating the treatment weight to the methods described above. Finally, we will seek to construct a classification rule based on predictors using techniques such as recursive partitioning and random forests using routines in R to identify groups of women defined by the exposure characteristics that have high probability of experiencing the adverse outcome.^50^

To identify predictors and characteristics of post-repair incontinence (Aim 2), we will first estimate the proportion of women who experience post-repair incontinence and the 95% confidence interval at multiple time points (e.g., 6m, 12m, 2y and 3y). Our primary analysis of predictors of persistent post-repair incontinence will focus on incontinence at 3 months, the time point by which incontinence resolvable through surgery will have resolved per expert opinion. We will first estimate bivariable relationships between each predictor and post-repair incontinence at this time point using multi-level mixed effects logistic regression modeling procedures in Stata to accommodate the clustered nature of our data.^51^ Subsequently, we will estimate one multivariable model to understand the independent and joint effects of patient, fistula, fistula repair, and post-repair characteristics on post-repair incontinence at 3m, integrating all independent variables that were associated with the outcome in bivariable analyses at conservative p < 0.1 and addressing correlation as described for Aim 1. To identify predictors of incident post-repair incontinence, we will assess incident post-repair incontinence and factors associated with time to incident post-repair incontinence using the survival analysis methods described for Aim 1. Finally, we will conduct analyses of binary predictors of interest employing propensity scores and seek to develop classification rules following the methods described for Aim 1.

#### Sample size.

The sample size for our longitudinal cohort study (up to n = 1000 women) was calculated to provide adequate power to detect a minimum difference in effect for patient-related, fistula-related, fistula-repair related, post-repair behaviors and exposures on risk of fistula repair breakdown or recurrence and incontinence of 20% (Aims 1 and 2) using the log-rank test for two-sample comparison of survivor functions (Aim 1) and the Pearson’s chi-squared two-sample proportions test (Aim 2). These effect differences were determined to be clinically significant based on expert opinion.

**Figure 4:**
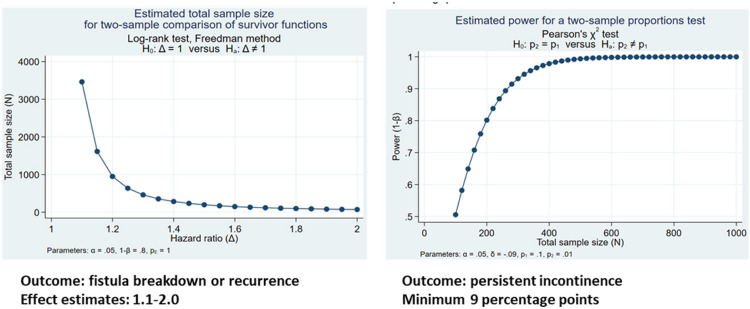
Sample size calculations for robust estimation of fistula breakdown or recurrence and persistent incontinence outcomes

Power calculations were developed using Stata’s power procedure, with values α = 0.05 and 1-β = 0.80.^51^ Prior research on fistula recurrence risk elsewhere suggests that factors of interest for our survival analyses (Aim 1) may have hazard ratios ranging from 1.0 to 3.4.^14^ Fig. 4 below illustrates the minimum sample size required for estimation of effect estimates ranging from 1.1 to 2.0 with parameters α = 0.05 and 1-β = 0.80, illustrating adequate power for two-sample comparison of survivor functions (i.e., time to fistula repair breakdown, time to fistula recurrence) with a sample size of 1000 for effect estimates (hazard ratios) of 1.2 or higher, illustrating a 20% or higher risk difference, accommodating some loss to follow-up. Research in other locations has reported a repair breakdown or recurrence rate of approximately 15%. With our target sample size of 1000, we anticipate being able to estimate this incidence with a range of precision of approximately 2.5% (i.e., between 12.8%−17.4%).

Sample size calculations for comparisons between risk factors of post-repair urinary incontinence at 6 and 12 months informing this analysis were estimated using the Pearson’s chi-squared two-sample proportions test with parameters α = 0.05 and 1-β = 0.80. The prior literature does not provide a good estimate of what range of potential risk elevation we are likely to see, so we have estimated risk differences of approximately 10 percentage points, across a range of possibilities. As shown in Fig. 4, for a potential comparison of 9 percentage points (e.g., from 1–10%) we achieve power of 0.80 at approximately 200 study participants. On the other end of the range (e.g., a comparison between 50% and 60%; Fig. 6), statistical power of 0.80 is achieved with a minimum sample size of 800 participants.

### Qualitative Component

Qualitative research with key stakeholders will be conducted to inform the development of feasible and acceptable intervention concepts targeting risk factors identified from our longitudinal cohort aims (Aims 1–2).

#### Study participants.

We will enroll approximately 80 individuals in total, including women with fistula, family members, community members, clinical and social service providers, and government. We will purposively sample ~ 40 longitudinal cohort participants to reflect study variability in region and adverse outcome experience. Other key stakeholders (~ 40) will be identified through discussion with study investigators, site leads and research assistants, and other clinical and social service providers for fistula in Uganda, to maximize variability in respondent region and clinical and patient support roles. Identified individuals will be invited to participate over the phone, email, or in-person and those who are interested and are willing to provide informed consent will be scheduled for an in-depth interview with a trained qualitative interviewer at a convenient time and private location. Informed consent for all respondents will be conducted in person, with written or thumbprint confirmation obtained, as appropriate. To respect the privacy and confidentiality of longitudinal cohort participants, permission will first be sought from the research participant before recruiting potential family member or peer qualitative participants.

#### Study procedures.

**Figure 5:**
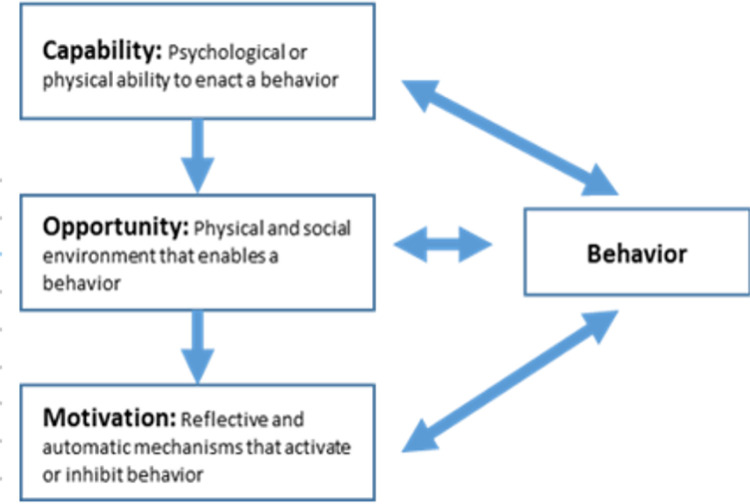
COM-B (‘capability’, ‘opportunity’, ‘motivation’, and ‘behavior’) model to inform intervention exploration for qualitative aim^54^

Based on our quantitative findings (Aims 1 and 2), literature, and expert clinical and contextual experience, the research team will develop a semi-structured and open-ended in-depth interview guide for key stakeholder interviews to obtain a nuanced understanding of their perspectives on feasible and acceptable potential intervention opportunities for addressing key risk and causal factors associated with adverse outcomes. Exploration of intervention possibilities with stakeholders may employ constructs from health behavior theories COM-B (‘capability’, ‘opportunity’, ‘motivation’, and ‘behavior’) model (Fig. 5) and the theoretical domains framework (TDF) for understanding individual and contextual issues, and the Consolidated Framework for Implementation Research (CFIR) for pre-implementation assessment of factors important to successful implementation (i.e., intervention characteristics, inner setting (characteristics of implementing organization), outer setting (features of the external context or environment), and implementation process (strategies or tactics for implementation setup or delivery).^52,53^ Interviews will be conducted in a private setting by an experienced qualitative interviewer and are anticipated to take 1–2 hours. Participants may be asked to respond iteratively as new data arises during the qualitative process. Interviews will be audio recorded and translated into English and transcribed.

#### Data analysis.

During the iterative interview and analysis process, we will combine COM-B,^54^ CFIR,^52^ and TDF analyses to identify a series of behavioral and implementation targets for each risk factor identified within our quantitative analysis, and for each of these we will 1) classify using the COM-B,^54^ 2) detail potentially modifiable determinants of behavior (e.g., barriers or facilitators) across CFIR^52^ domains, 3), list the theoretical domain and techniques for behavior change using the TDF, and 4) develop and assess potential implementation strategies across multiple actors to achieve the desired change.

Further qualitative data analysis will follow a 2-stage systematic process.^55^ The first stage will involve data coding and classification by reviewing the transcripts for potential conceptual categories, using the in-depth interview guide. Two types of codes will be employed: deductive and inductive/emergent. First, deductive codes that represent expected influences will be applied to the data; these will be taken from the existing literature and the theoretical orientation of the interview guide (i.e., COM-B, TFR and CFIR construct list). Next, inductive codes that emerge organically from the data will represent themes that were not expected by the researchers. Emergent themes will be identified based on recurrence rate and on similarities and differences noted across the texts. A codebook will be developed from the themes that will include a detailed description of each code, code inclusion and exclusion criteria, and examples of the code in use. Coded data will be analyzed to describe the different dimensions and commonalities of each theme, their distribution across socio-demographic variables, and the patterns and linkages between themes. Comparisons will be made to detect divergent views among participants and to contrast observations by sample population characteristics and type of key stakeholder.

#### Sample size.

The qualitative sample size was selected on the basis of our prior experience with thematic saturation; however, final sample size will be determined through iterative assessment of theme saturation as data are collected across different participant types.^56^

## Discussion

In this study, we seek to estimate the contribution of a broad range of potential risk factors of key adverse outcomes following genital fistula repair: fistula repair breakdown or recurrence, persistent urinary incontinence, and incident urinary incontinence. Our study overcomes several key limitations to the current evidence base through recruiting a sample size capable of robust assessment, employing a longitudinal design to enable evaluation of time-varying contributors and a longer time frame through extending participant follow-up. We also focus on a broad range of patient, clinical, and behavioral characteristics to best inform the development of relevant clinical care interventions, paying particular attention to those that are modifiable without excluding those currently considered unmodifiable, which will guide us to identifying subgroups at highest risk for ensuring care engagement and influence subsequent research priorities.

The results of this study will inform key intervention targets for integration into clinical and counseling interventions to mitigate these risks and ensure women’s high quality of life following genital fistula repair. We engage key stakeholders (e.g., women with fistula, family members, community members, clinical and social service providers, government) in interpretation of our findings and strategy development activities to improve the translation of our quantitative findings into feasible and acceptable intervention possibilities.

The next steps of this research program include intervention development, employing the strategies arising from the proposed research, assessment of acceptability and feasibility, and testing for effectiveness. This broader program is likely to result in tangible recommendations and intervention strategies for improving women’s health and wellbeing following genital fistula repair in the short and long term, allowing them to move on to healthy and productive lives. This work is an important corollary to existing efforts to increase access to genital fistula surgery among the estimated 500,000–2 million women currently living with this condition.^1,57^

In addition to informing an important evidence gap, establishing a large longitudinal cohort such as this represents an important opportunity to develop an important resource for investigating other important research questions on the period following genital fistula repair, including those focused on other physical concerns and psychosocial trajectories and outcomes, and we will encourage and support investigation in these areas through professional development among research team members, collaborating with other researchers, and developing capacity through the involvement of trainees in our project.

### Dissemination plan

Sharing of findings generated by this study is an essential part of our research activities and will be carried out in several different ways. The research team’s own academic and community settings offer many opportunities for learning and sharing. As the study progresses, we will arrange briefing meetings with Ministry of Health officials and other interested stakeholders, to keep them informed of our study progress. We will hold an in-person study dissemination meeting at the end of the study including important Ugandan key stakeholders and media. We will disseminate the study results through presenting at relevant global maternal health conferences. We will take the opportunity to disseminate our findings at other important outlets, including invited meetings and webinars within the fistula and maternal health communities. We will also share our findings with the international community through peer-reviewed publications. We will make our results available to the community of scientists interested in genital fistula and more broadly in maternal and reproductive health. At the end of the study and after the manuscripts addressing the primary analyses are published, the investigators will allow other interested investigators who were not part of the primary study to collaborate on other questions of mutual interest that fit within the overall scope of the proposed implementation research. We will make the de-identified data and associated documentation available to users through Dryad, an open data publishing platform, under a data sharing agreement.

## Figures and Tables

**Table 1:** Study measurements and timing of data capture for longitudinal cohort

Outcome Variables							
Category	Measure Description	Data Source^a^	Data Collection Timeline				
			Bl	2w	6w	3m	6–36m, quarterly
Fistula repair breakdown or recurrence (Aim 1)	Reopening of the fistula following repair, prior to complete healing, or *de novo* fistula occurrence. Confirmed by positive methylene blue dye test or other method.	MR	●	●	●	●	●
Post-repair urinary incontinence (Aim 2)	Urinary incontinence with confirmed fistula closure	MR	●	●	●	●	●
**Predictor Variables**							
Patient-related: study participant characteristics, potentially important biological and social risk factors for poor health.							
Sociodemographics	Age, educational attainment	PQ	●				
	Income, assets, food security^58^	PQ	●				●
Obstetric history	Parity, pregnancy outcomes (prefistula, during-fistula), time since fistula development	PQ, MR	●				
Health status	Nutritional status^b^	MR	●	●	●	●	●
	Co-morbidities,^c^ urinary tract infection, functional health,^d^	PQ, MR	●	●	●	●	●
Fistula-related: selected characteristics illustrate fistula severity and physical burden of fistula.							
Fistula characteristics	Size, location, fistula etiology^e^, fistula type^f^, VVF grade^g^, vaginal scarring, bladder capacity, urethral length, bladder neck involvement, other urogyn diagnoses.	MR	●				
Fistula history	Time since fistula development, etiology, symptoms^h^, number of previous repairs, treatments.	PQ, MR	●				
Fistula repair-related: measures include provider training, procedural decisions, and complications.							
Repair procedure	Surgical route^i^; layers, suture type; anesthetic type; sling and tension; flap use, graft use and type, prophylactic antibiotic use, catheterization^j^	MR, PS	●				
Quality of care	Person-centered care	PQ	●				
Provider characteristics	Surgeon, surgical level and experience, repair center	PS	●				
Repair complications	Bleeding, infection, leakage, pain (48hrs+), catheter blockage, other	MR, PS	●				
Post-repair behaviors and exposures: variables capture physical and sexual risks.							
Physical activity and trauma	Moderate and vigorous activity, peak and long-term weight lifting^k^, physical violence^l^	PQ	●	●	●	●	●
Sexual activity and fertility	Post-surgical resumption of sexual activity, frequency of sexual activity, sexual satisfaction^m^, fertility intentions, menstruation, contraceptive use.	PQ	●	●	●	●	●
Lifestyle	Dietary quality, alcohol^n^, tobacco^o^, and caffeine consumption^p^; medical and traditional medical care.	PQ	●	●	●	●	●
Pregnancy-related factors: pregnancy-related factors below may contribute to risk of adverse outcomes through biological or social mechanisms.							
Pregnancy-related health	Chronic and pregnancy-related co-morbidities^q^, timing of pregnancy	PQ, MR	●	●	●	●	●
Antenatal care	ANC initiation, timing, frequency and location, birth planning	PQ, MR	●	●	●	●	●
Delivery-related	Gestational age at delivery, delivery mode^r^, birth attendant, length of labor	PS, MR	●	●	●	●	●
Delivery complications	Prolonged/obstructed labor, hemorrhage, other.	PS, MR	●	●	●	●	●
**Other Variables**: selected characteristics are important for a broader understanding of women’s recovery from fistula and repair.							
Psychosocial health	Reintegration^s^, quality of life^t^, depression^u^ anxiety^v^, self-esteem^w^ stigma^q^, social support^y^, and relationship quality^z^, PTSD^aa^	PQ	●	●	●	●	●
Sexual function	Sexual function and satisfaction^bb^	PQ	●	●	●	●	●
Adjunct service receipt	Receipt of any psychological, physical, social, or economic services or assistance, and dose.	PQ	●	●	●	●	●
Empowerment	Economic control^cc^, patient knowledge	PQ	●	●	●	●	●

*Data Sources include Patient Questionnaire (PQ), Medical Record (MR) through standardized form (with provider follow-up), and Provider Survey (PS), Urodynamic Testing (UT). Detailed measure descriptions:* ^b^Body mass index, anemia ^c^Diabetes, malaria, HIV, hypertension, anemia, pre-eclampsia, UTI ^d^WHODAS 2.0 Short form;^59 e^Obstetric, iatrogenic, traumatic; ^f^VVF, RVF, VCVF, left/right ureteric, utero-vesical; ^g^Waaldjik & Goh classification; ^h^ICIQ-UI-SF (urinary incontinence);^60,61^ ICIQ-UI-SF – modified for fecal incontinence; ^i^Vaginal vs. abdominal; ^j^Route and number of days; ^k^International Physical Activity Questionnaire – short form;^62 l^Type and intensity;^63,64 m^PISQ-IR (Pelvic Organ Prolapse/Urinary Incontinence Sexual Questionnaire, IUGA-Revised);^65^ Couple Functionality Assessment Tool (sexual communication);^66^ Couple Sexual Satisfaction Scale;^67 n^Modified Alcohol Use Disorders Identification Test (AUDIT);^68 o^Modified Global Adult Tobacco Survey (GATS);^69 p^Modified Caffeine Consumption Questionnaire (CCQ);^70 q^diabetes, hypertension, preeclampsia, malaria, UTI, anemia; ^r^vaginal, elective cesarean, emergency cesarean; ^s^Post-repair fistula reintegration instrument;^71 t^WHO QOL BREF;^72^ uPatient Health Questionnaire-9;^73 v^Generalized Anxiety Disorder-7;^74 w^self-esteem scale;^75 x^ Adapted fistula-related stigma measure;^76 y^Adapted Multidimensional Scale of Perceived Social Support;^77–79^ zCommittment, trust, communication, relationship satisfaction, intimacy and treatment by partner;^80^ Triangular Scale of Love, Emotional Intimacy Scale;^81^ Couple Satisfaction Index;^82^ 8-item Dyadic Trust Scale;^83^ Inclusion of Other in the Self (IOS) Scale;^84^ 3-item mutually constructive communication (MCC) subscale of the Communications Patterns Questionnaire;^85 aa^City Birth Trauma Scale Version 2.0 2018;^86 bb^PROMIS full profile 2.0 sexual function and satisfaction;^87 cc^Household Decision Making Power.^88^

## Data Availability

The datasets to be generated during and analysed during the current study will be de-identified and made publicly available after the study is complete and all planned analyses are achieved.

## List Of Abbreviations

COM-B: capability, opportunity, motivation, and behavior framework
CFIR: consolidated framework for implementation research
M: month
NGO: non-governmental organization
TDF: theoretical domains framework
Y: year
